# Tarlatamab in Relapsed SCLC With Idiopathic Pulmonary Fibrosis: A Case With Comparative DLL3 Expression Analysis: Case Report

**DOI:** 10.1016/j.jtocrr.2026.101052

**Published:** 2026-07-14

**Authors:** Minori Takamura, Kentaro Tamura, Risa Sugito, Naoko Takahashi, Shiko Honma, Masahiro Yoshida, Hiroshi Wakui, Hitomi Sekiguchi, Ryusuke Kizawa, Susumu Saito, Sachi Matsubayashi, Ayu Kiritani, Keitaro Okuda, Hirofumi Utsumi, Saburo Ito, Shunsuke Minagawa, Takanori Numata, Hiromichi Hara, Jun Araya

**Affiliations:** a Division of Respiratory Diseases, Department of Internal Medicine, The Jikei University School of Medicine, Tokyo, Japan; b Department of Pathology, The Jikei University School of Medicine, Tokyo, Japan

**Keywords:** Small cell lung cancer, Idiopathic pulmonary fibrosis, Tarlatamab, Delta-like ligand 3, Interstitial lung disease, Case report

## Abstract

There is no established treatment for relapsed SCLC in patients with interstitial lung disease. We report the case of a 67-year-old man with idiopathic pulmonary fibrosis (IPF) receiving nintedanib who developed a postoperative relapse of SCLC. After cisplatin plus etoposide therapy, the patient developed an acute exacerbation of IPF, which improved with corticosteroid therapy. However, pleural dissemination and pulmonary metastases progressed, leading to the initiation of tarlatamab. During follow-up, transient ground-glass opacities appeared around pulmonary metastases. These opacities resolved, and tumor shrinkage was observed. The patient achieved a partial response without new toxicities, and the therapy was ongoing. Immunohistochemical analysis using an anti–delta-like ligand 3 antibody (SP347) reported strong expression in tumor cells but no detectable expression in adjacent normal or fibrotic lung tissue. This finding provides a possible biological rationale. This case suggests that tarlatamab may be a potential therapeutic option in selected patients with relapsed SCLC complicated by IPF.

## Introduction

Effective treatment options for relapsed SCLC remain limited. Tarlatamab, a delta-like ligand 3 (DLL3)–targeting bispecific T-cell engager, has reported promising efficacy in relapsed SCLC.^1^

Interstitial lung disease (ILD) complicates treatment decisions in SCLC.^2^ However, the safety and radiologic interpretation of pulmonary findings during tarlatamab in patients with ILD remain unclear.

Here, we report a case of relapsed SCLC with idiopathic pulmonary fibrosis (IPF) treated with tarlatamab, in which transient ground-glass opacities (GGOs) were observed and interpreted as peritumoral inflammatory changes rather than ILD exacerbation, supported by comparative DLL3 expression analysis.

## Case Presentation

**Figure 1:**
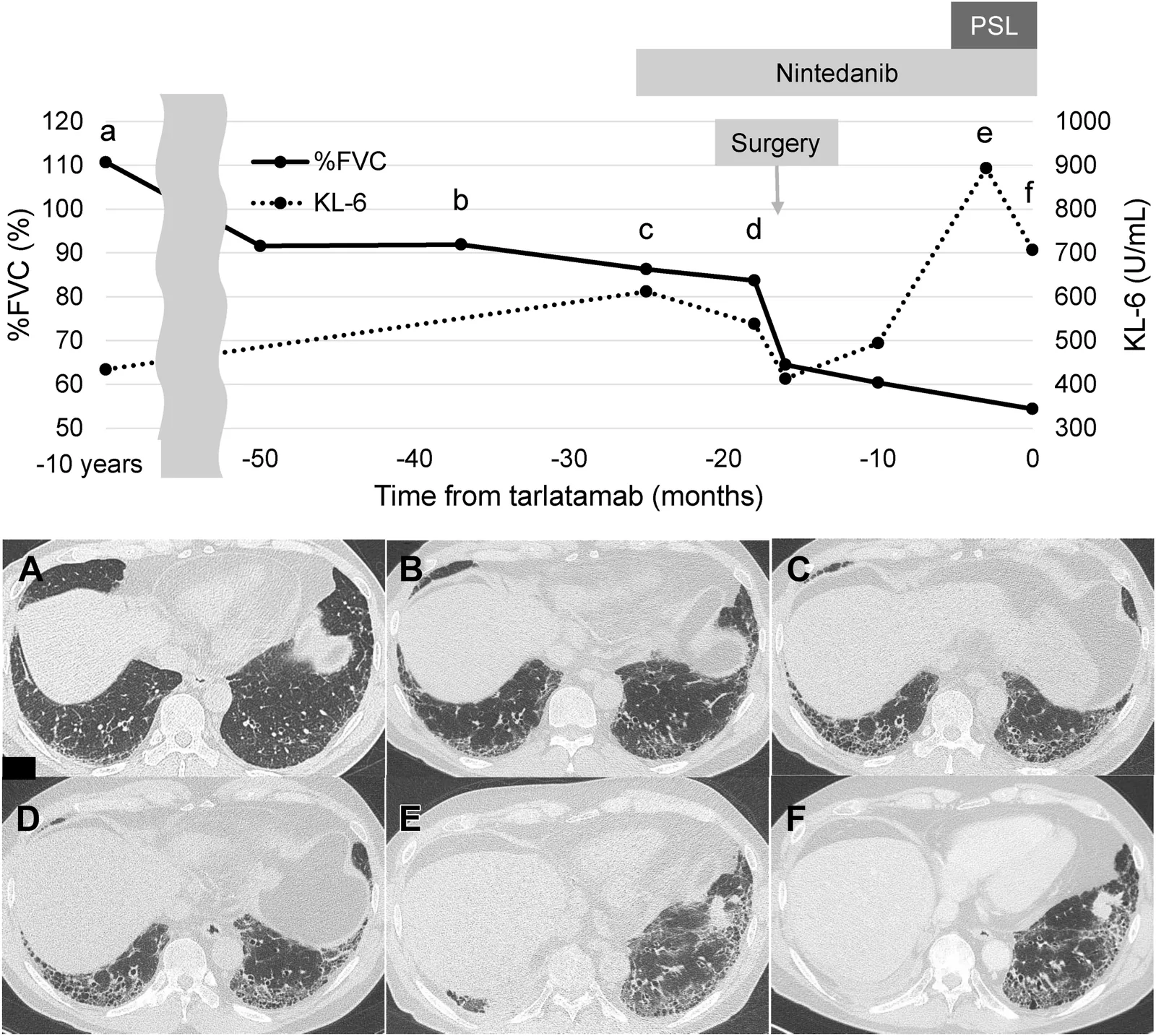
Clinical course of idiopathic pulmonary fibrosis before tarlatamab treatment. Chest HRCT obtained approximately 10 years before tarlatamab revealed subpleural reticulation in the lower lobes (*A*). Fibrosis and pulmonary function gradually worsened (*B*: 37 mo and *C*: 25 mo before tarlatamab). After nintedanib was initiated, disease progression was suppressed (*D*: 18 mo before tarlatamab administration). The patient subsequently underwent right lower lobectomy and was diagnosed with limited-stage SCLC (pT1cN2aM0, stage IIB). Acute exacerbation of IPF occurred 3 months before tarlatamab, with new ground-glass opacities and a decline in pulmonary function (*E*), which improved after corticosteroid treatment (*F*). % FVC, percent-predicted forced vital capacity; HRCT, high-resolution computed tomography; IPF, idiopathic pulmonary fibrosis; KL-6, Krebs von den Lungen-6; PSL, prednisolone.

A 67-year-old man with a smoking history had been followed at our hospital for IPF for 10 years. Nintedanib was initiated 2 years before tarlatamab and slowed the progression of fibrosis (Fig. 1*A*, *B*, *C*, and *D*). After 6 months, a solid nodule was detected in the right lower lobe. The patient underwent a right lower lobectomy, and pathologic examination confirmed limited-stage SCLC, pT1cN2aM0, stage IIB, according to the ninth edition of the TNM classification. The background lung had usual interstitial pneumonia (UIP). The patient received adjuvant cisplatin and etoposide. Five months after completing adjuvant chemotherapy, pleural dissemination and pulmonary metastases were detected, indicating a sensitive relapse of SCLC. He subsequently received four cycles of cisplatin plus etoposide, which resulted in stable disease.

**Figure 2:**
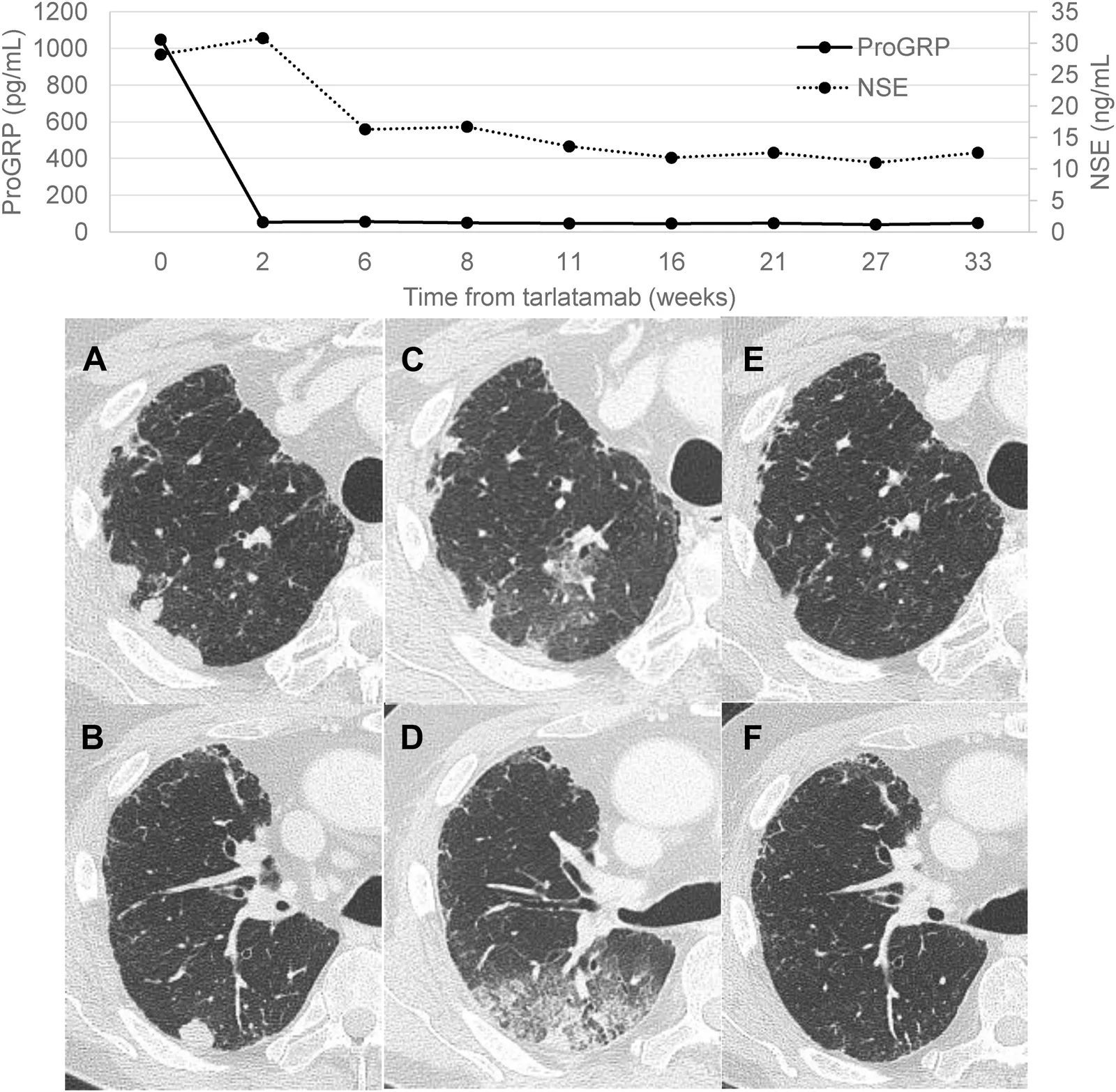
Treatment response to tarlatamab. Pretreatment HRCT reveals pleural dissemination and pulmonary metastases in the right lung (*A* and *B*). Two weeks after tarlatamab administration, HRCT reveals GGOs surrounding pulmonary metastases, accompanied by tumor shrinkage (*C* and *D*). After 33 weeks (8 mo) from tarlatamab administration, pleural dissemination in the right lung had resolved (*E* and *F*). Serum ProGRP and NSE levels normalized (upper limits of normal at our institution: ProGRP, 80.9 pg/mL; NSE, 16.3 ng/mL). GGO, ground-glass opacities; HRCT, high-resolution computed tomography; NSE, neuron-specific enolase; ProGRP, progastrin-releasing peptide.

However, 1 month after the final course of cisplatin and etoposide, the patient developed an acute exacerbation of IPF based on clinical and radiologic findings (Fig. 1*E*). He was treated with corticosteroids, with improvement, and stability was maintained after tapering prednisolone to 10 mg/d. Computed tomography (CT) revealed honeycombing with traction bronchiectasis in the left basal region and progression of fibrosis, consistent with UIP, along with progression of pleural dissemination and pulmonary metastases (Fig. 1*F*, Fig. 2*A* and *B*).

Tarlatamab therapy was initiated after obtaining informed consent. The patient developed grade 1 cytokine release syndrome, which was managed with tocilizumab. Grade 3 anorexia without dysgeusia was observed. A chest and abdominal CT scan was performed to evaluate for disease progression, acute exacerbation of IPF, and infection. As the patient had no gastrointestinal symptoms, such as nausea or abdominal pain, further gastrointestinal evaluation was deferred. The anorexia resolved spontaneously within 1 week. A chest CT incidentally revealed localized GGOs adjacent to the tumor, consistent with peritumoral inflammatory changes (Fig. 2*C* and *D*). These opacities were confined to the peritumoral region without diffuse distribution. The patient had no respiratory symptoms, and serum Krebs von den Lungen-6 and surfactant protein D levels remained stable, arguing against acute exacerbation of IPF or drug-induced pneumonitis. The opacities resolved spontaneously with tumor shrinkage without additional treatment (Fig. 2*E* and *F*). The best overall response was a partial response (Fig. 2*E* and *F*). Currently, 8 months after the initiation of treatment, the patient remains on tarlatamab treatment without evidence of IPF exacerbation or other significant adverse events. A nodular lesion in the left lower lobe, which had been present before tarlatamab initiation, had gradual enlargement over time, unlike the other lesions that responded to treatment. The lesion was later resected and pathologically diagnosed as squamous cell carcinoma, consistent with a metachronous second primary lung cancer (Supplementary Fig. 1).

**Figure 3:**
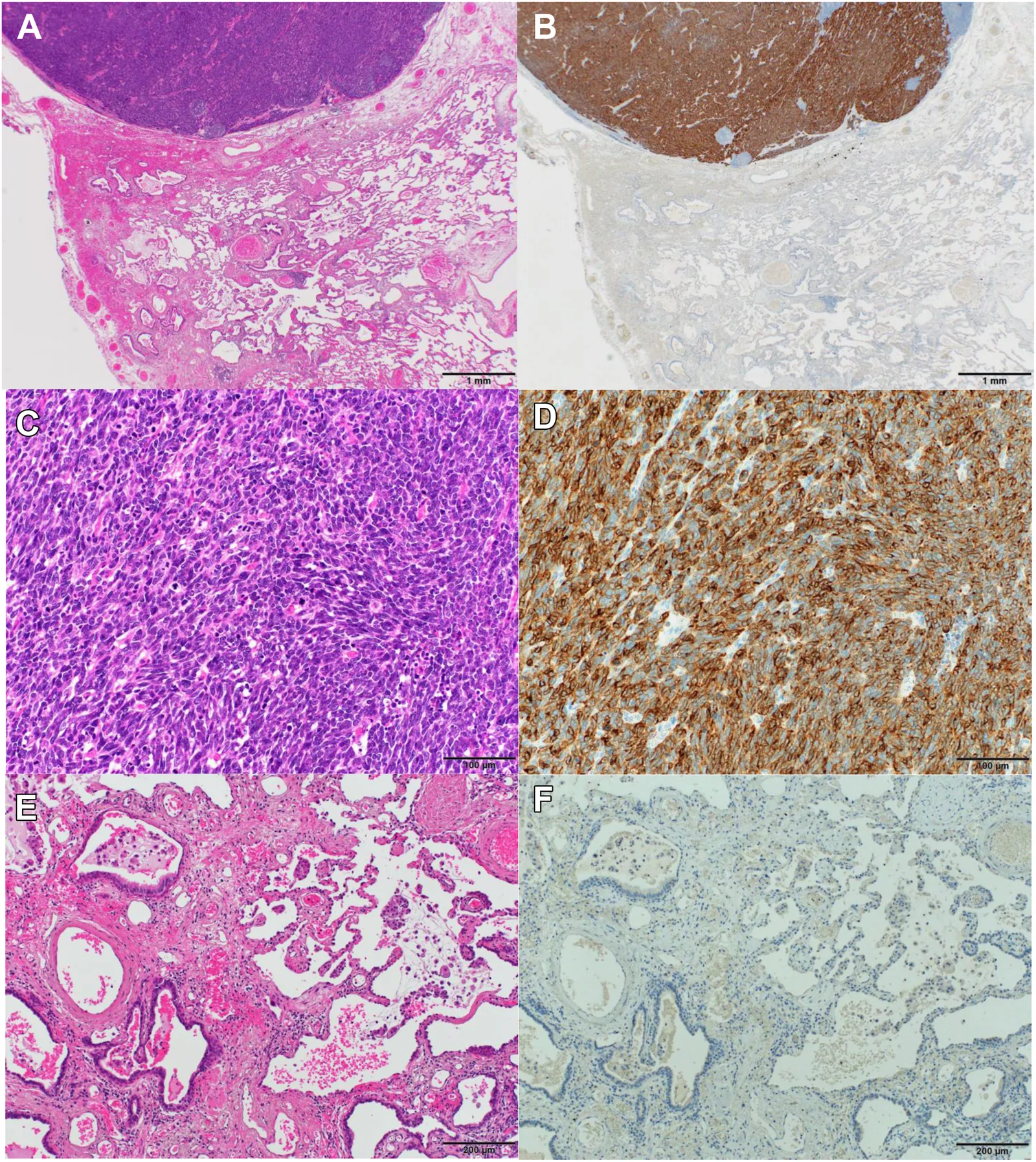
DLL3 immunohistochemistry in the resected specimen. Representative histologic and immunohistochemical findings from the resected lung. Low-power views reveal SCLC adjacent to background lung parenchyma with interstitial changes (*A*: hematoxylin and eosin staining; *B*: DLL3 immunohistochemistry). Diffuse granular cytoplasmic DLL3 expression with moderate-to-strong (approximately 2–3+) intensity was observed in tumor cells, whereas no expression was detected in the surrounding non-neoplastic alveolar epithelium or interstitial lung lesions consistent with UIP. High-power views of the tumor reveal typical SCLC morphology and strong DLL3 expression (*C*: hematoxylin and eosin staining; *D*: DLL3 immunohistochemistry). Representative areas of UIP reveal fibrotic remodeling without DLL3 expression (*E*: hematoxylin and eosin staining; *F*: DLL3 immunohistochemistry). Scale bars: a, b = 1 mm; c, d = 100 μm; e, f = 200 μm. DLL3, delta-like ligand 3; UIP, usual interstitial pneumonia.

Immunohistochemical staining for DLL3 was performed using an anti-DLL3 antibody (clone SP347; Roche Diagnostics) on the resected right lower lobectomy specimen.^1^ DLL3 reported strong and diffuse granular cytoplasmic expression with moderate-to-strong (approximately 2–3+) intensity in SCLC tumor cells, based on the scoring approach reported by Serrano et al (Fig. 3*A*, *B*, *C*, and *D*).^3^ In contrast, no detectable DLL3 expression was observed in adjacent normal alveolar epithelium or in areas of histologically confirmed UIP (Fig. 3*A*, *B*, *E*, and *F*).

## Discussion

We report a case in which tarlatamab was administered to a patient with relapsed SCLC and IPF after an acute exacerbation.

In the phase 2 DeLLphi-301 trial of tarlatamab, the incidence of pneumonitis was reported to be 0.8%, and the toxicity was generally mild.^1^ One possible explanation for the relatively low pulmonary toxicity of tarlatamab compared with that of immune checkpoint inhibitors is the distinct expression profile of its target, DLL3. DLL3 is highly expressed on SCLC cells and is minimally expressed in normal tissues.^4^ In our case, immunohistochemical analysis reported strong DLL3 expression in SCLC tumor cells, but no detectable expression was observed in adjacent normal alveolar epithelium or in areas of UIP in this specimen. Tarlatamab redirects cytotoxic T cells toward DLL3-expressing tumor cells. This mechanistic difference, together with the absence of DLL3 expression in fibrotic lung tissue, may partly explain the limited pulmonary toxicity observed in this case. Publicly available transcriptomic data sets (GEO: GSE150910 and Chan et al.^5^) reveal that DLL3 expression was enriched in SCLC cells but low in nonmalignant lung tissues, including IPF lungs (Supplementary Fig. 2 and Methods).

In this case, transient GGOs appeared around pulmonary metastases after tarlatamab administration. Given their localized distribution and the absence of clinical deterioration, these GGOs were considered peritumoral inflammatory changes, consistent with a localized immune-mediated reaction in tumor-bearing areas. The gradual resolution of these opacities, together with ongoing tumor shrinkage, further supports this interpretation. In addition, radiologic-pathologic correlation of the GGOs was not available, and the interpretation remains inferential.

However, this case report does not imply that tarlatamab can be safely administered to all patients with SCLC and ILD. Careful patient selection, thorough informed consent, and close monitoring are essential before the treatment is initiated, particularly in patients with underlying ILD. In addition, our observation of absent DLL3 expression in IPF tissue is limited to a single specimen and may be influenced by spatial or temporal heterogeneity. Further investigation of DLL3 expression in fibrotic lung tissue across a larger cohort is warranted.

Management of cytokine release syndrome (CRS) also required special consideration in this patient with underlying IPF. Because the patient had underlying IPF with limited pulmonary reserve, we were concerned that even mild CRS could trigger respiratory deterioration. In addition, the patient had already been receiving prednisolone 10 mg/d for IPF management, and we sought to avoid further corticosteroid escalation because of concerns regarding the attenuation of antitumor immune activity and potential steroid-related pulmonary complications. Therefore, tocilizumab was administered at an early stage of CRS.

## Conclusion

Tarlatamab may be a therapeutic option for selected patients with relapsed SCLC complicated by IPF. The absence of DLL3 expression in fibrotic lung tissue may partly explain its limited pulmonary toxicity.

## CRediT Authorship Contribution Statement

**Minori Takamura:** Conceptualization, Investigation, Data curation, Visualization, Writing – original draft.

**Kentaro Tamura:** Conceptualization, Investigation, Data curation, Formal analysis, Visualization, Writing – original draft, Writing – review & editing.

**Risa Sugito:** Investigation, Writing – review & editing.

**Naoko Takahashi:** Investigation, Writing – review & editing.

**Shiko Honma:** Investigation, Formal analysis, Writing – review & editing.

**Masahiro Yoshida:** Formal analysis, Writing – review & editing.

**Hiroshi Wakui:** Writing – review & editing.

**Hitomi Sekiguchi:** Writing – review & editing.

**Ryusuke Kizawa:** Writing – review & editing.

**Susumu Saito:** Writing – review & editing.

**Sachi Matsubayashi:** Writing – review & editing.

**Ayu Kiritani:** Writing – review & editing.

**Keitaro Okuda:** Writing – review & editing.

**Hirofumi Utsumi:** Writing – review & editing.

**Saburo Ito:** Writing – review & editing.

**Shunsuke Minagawa:** Writing – review & editing.

**Takanori Numata:** Writing – review & editing.

**Hiromichi Hara:** Writing – review & editing.

**Jun Araya:** Writing – review & editing and supervision.

## Disclosure

The authors declare no conflict of interest.
