# Supplementary material for: Tarlatamab in Relapsed SCLC With Idiopathic Pulmonary Fibrosis: A Case With Comparative DLL3 Expression Analysis: Case Report
Source: JTO Clin Res Rep. 2026 Jul 14;7(10):101052. doi: 10.1016/j.jtocrr.2026.101052 (PMC13571922; doi:10.1016/j.jtocrr.2026.101052)
Supplement: Supplementary file 1 [file mmc1.docx]

**Supplementary Methods**

Transcriptomic data were obtained from publicly available datasets. Bulk RNA sequencing data were retrieved from the Gene Expression Omnibus (GEO; accession number: GSE150910) and used to compare DLL3 expression between idiopathic pulmonary fibrosis (IPF) and control lung tissues.

Single-cell RNA sequencing data were obtained from a previously published dataset (Chan et al.). Cell type annotations were based on those provided in the original study. DLL3 expression across annotated cell types was analyzed and visualized.

Statistical comparisons between groups were performed using the Wilcoxon rank-sum test.
